# Patients‐in‐waiting or chronically healthy individuals? People with elevated cholesterol talk about risk

**DOI:** 10.1111/1467-9566.12866

**Published:** 2019-01-22

**Authors:** Mikko Jauho

**Affiliations:** ^1^ Centre for Consumer Society Research Faculty of Social Sciences University of Helsinki Helsinki Finland

**Keywords:** cardiovascular disease, cholesterol, chronic illness, lay experiences, lifestyle, risk

## Abstract

Risk adopts an ambiguous position between health and illness/disease and is culturally salient in various health‐related everyday practices. Previous research on risk experience has mostly focused on the illness/disease side of this risk ambiguity. Persons at risk have typically been defined as patients (of some kind) and their condition as a form of proto‐illness. To allow for the cultural proliferation of health risk and to account for the health side of risk ambiguity, I chose to focus on elevated cholesterol, a condition both intensely medicalised and connected to the everyday practice of eating, among participants (n = 14) recruited from a consumer panel and approached not as patients, but as individuals concerned about their cholesterol. Utilising the biographical disruption framework developed by Bury, I show how the risk experience of my participants differed from the chronic illness experience. Instead of patients‐in‐waiting suffering from a proto‐illness, they presented themselves as ‘chronically healthy individuals’ (Varul 2010), actively trying to avoid becoming patients through a responsible regimen of personal health care. The results call for a more nuanced approach to the risk experience, which accounts for both sides of the risk ambiguity.

## Introduction

Medicine, health promotion and personal health care today are increasingly oriented towards health risks. Accounting for the post‐Second World War rise of chronic degenerative diseases such as lung cancer and coronary heart disease created a novel medical rationality that focused on population distributions of risk factors (Berlivet 2005, Parascandola 2004, Rothstein 2003, Talley *et al*. 2004). These are defined as attributes, characteristics or exposures of an individual (as a member of a population group) that increase the likelihood of developing an injury or disease (WHO 2018). Due to this probabilistic logic, medicine no longer only identifies and treats present signs and symptoms of pathology, but also assesses and controls virtual conditions (Armstrong 1995), or what Rosenberg (1997) calls protodiseases.

The epidemiological rationale underpinning the risk factor approach greatly expands the scope of medicine. Although the number of people exhibiting signs and symptoms of illness is in principle finite, everyone is more or less at risk of some illness. Moreover, probabilistic risk factor prevention requires significant target groups: to prevent one endpoint, a considerably larger number of individuals needs to be treated and risk factor distributions dictate that most cases come from the groups at average risk, not high risk (Rose 1981). The risk factor approach thus has an inherent expansive tendency, which is further exacerbated by a tendency to sink the thresholds signifying treatable conditions (Greene 2007) and by introducing novel disease concepts such as pre‐diabetes or pre‐hypertension (Kreiner and Hunt 2014).

Significant for the current configuration of this ‘mass health’ (Dumit 2012) is the fact that the risk factors singled out by chronic disease epidemiology were typically individual‐based, either physiological markers amenable to modification (blood pressure, blood cholesterol) or aspects of behaviour (smoking, exercising), whereas social and structural variables retained a subordinate role (Giroux 2013, Oppenheimer 2005). This has highlighted individual lifestyles as the primary target of illness prevention and health promotion (Hansen and Easthope 2007). The focus on lifestyle choices has had a wide cultural resonance in industrialised countries. A number of mundane everyday practices such as eating or exercising are structured by considerations concerning the health effects of personal choices. These choices are often made on the market, where commercial actors offer a myriad of products and services for a healthy lifestyle (Bunton and Burrows 1995). As a consequence, health has become a matter of personal responsibility and illness a sign of moral failure in the endeavour of personal health care (Hughes 1994, Lupton 1993).

This ‘spillover’ into everyday life and consumption is especially salient in the case of cholesterol, the topic of this article. Elevated cholesterol has been identified as one of the key risk factors for CVD, especially coronary heart disease and stroke. While cholesterol levels are partly determined by genes, in the causation of CVD emphasis has been placed on behavioural factors, especially diet. A diet high in saturated fats and low in unsaturated fats is considered the prime factor for elevated cholesterol (or a poor LDL/HDL cholesterol ratio). This dietary doctrine has been adopted by nutrition guidelines and steers CVD‐related health policy measures in Western countries (Garrety 1997, Jensen 1994, Madarász 2010, Nestle 2007), including Finland (Kokko and Räsänen 1997). It has also become a truism in the popular understanding of nutrition (La Berge 1998). Saturated fat or fatty foods are routinely mentioned by consumers as the main ‘bad’ to be avoided in a healthy diet, alongside sugar, salt, fast‐food and processed foods (e.g. Paquette 2005, Povey *et al*. 1998). Food producers have seized the opportunity and market a wide array of low‐fat and cholesterol‐lowering products. Thus, the medical risk of elevated cholesterol figures prominently in the way people understand health and consume food today.

This article discusses the implications of the shift to risk‐driven mass health for individuals managing their health and uses elevated cholesterol as an example. More specifically, I approach risk from the perspective of everyday healthcare practices, allowing for its spread from the medical into other realms. This is reflected in the recruitment of the participants, which were from a consumer panel, not from a medical context. Earlier studies on risk experience have typically modelled it on the chronic illness experience and centred on patienthood. Consequently, it felt appropriate to analyse the interviews utilising the most influential framework for the experience of chronic illness, Bury's (1982, 1991) notion of biographical disruption. Surprisingly, my participants’ experience did not conform to this framework or key points of previous research on the (cholesterol) risk experience. Rather, they sought to distance themselves from patient status. The conclusion reiterates the importance to study risk ambiguity from both the illness/disease and health side.

## The experience of risk: patients‐in‐waiting?

Risk does not fit easily into the established distinctions between health, illness and disease (e.g. Kleinman 1988). As health risks are future‐oriented and probabilistic, they do not necessarily create feelings of being sick, an illness experience. Neither do they as such constitute a disease process to be diagnosed and treated. For example elevated cholesterol does not produce symptoms and cannot be diagnosed without testing (Angus *et al*. 2005, Hoel Felde 2010). Although the pathological effect of high cholesterol, the thickening of the arterial walls and narrowing of blood vessels, could in principle be measured, this has not been established as a standard clinical procedure. Yet risks have become intensely medicalised and are increasingly becoming objects of treatment. Elevated cholesterol is a case in point: hyperlipidaemia is classified as both a risk marker for CVD and a separate disease, and duly medicated. Large portions of the adult population are on preventive cholesterol medication that they will take for the rest of their lives (Dumit 2012). Persons at risk thus occupy an ambiguous position somewhere between health and illness/disease.

Previous research has introduced a number of concepts to address this specific position or the ‘technoscientific illness identity’ (Sulik 2009) of individuals at risk of illness. Such conceptual work has been fuelled by recent interest in the genomic origins of disease, but those at risk of conditions without an identified genetic component face similar issues (Davison *et al*. 1994). Both groups of individuals have to live with risk and negotiate uncertainty.

Crawford (1980), in his discussion on healthism, introduced the ‘potential sick role’ concept to describe the social position of individuals facing uncertainty before receiving test results or a firm diagnosis. Social obligations are less clearly defined in this at‐risk status than in acute illnesses, the original reference point of Parsons’ concept (Kenen 1994). Greaves (2000) called ‘partial patients’ those who do not feel ill or disabled but have been diagnosed with having or as being at risk of developing a disease, including patients who are convalescent, in remission, or survivors of a medical condition. The experience of risk is thus incorporated into a larger category, encompassing presently asymptomatic individuals with varying levels and histories of medical contact. According to Gillespie (2015), the social experience of life with an identified health risk – in his case, elevated cholesterol or PSA‐levels – resembles the experience of proper (chronic) illness, thus justifying his notion of ‘proto‐illness’ (cf. Rosenberg 1997).

Similar themes have been addressed in the context of the effects of predictive genetics on individual experiences and relations to family and kin. Genomic knowledge has been diagnosed with the capacity to create 'pre‐symptomatic persons' (Konrad 2003) and 'potentially sick, potentially vulnerable' individuals (Kenen 1994). In their study of healthy women with a family history of breast or ovarian cancer, Kenen *et al*. (2003) explicitly discuss the condition in the chronic illness framework, introducing the concept of ‘chronic risk’ to describe the overlap between the chronic illness and risk experiences. In a similar vein, Finkler (2001: 241) calls individuals at a genetic risk of breast cancer ‘perpetual patients’. They are people ‘who [have] entered the medical stream despite the fact that [they are] healthy’. Although she does not explicitly refer to the chronic illness experience, the feelings of and actions taken by her interviewees focus on various aspects associated with patienthood, such as anxiety and medical contact.

Summing up the research field, Timmermans and Buchbinder (2010: 409) have suggested ‘patients‐in‐waiting’ as ‘an overarching concept to elucidate common experiences among people trapped between a state of sickness and health characterised by uncertainty about disease’ in relation to both genomic and non‐genomic understandings of disease. Their starting point is the concept of diagnostic uncertainty, which has been used in studies related to chronic pain and contested illnesses. According to them, parents of newborns screened for genetic conditions, people undergoing genetic susceptibility testing, people suffering from proto‐disease states such as elevated cholesterol and children with suspected developmental disabilities face similar uncertainty. The experience of risk is thus assimilated into a larger class of conditions facing uncertainty in a medical context. Such patients‐in‐waiting ‘inhabit a liminal state between normalcy and pathology, imposed by medical screening and testing technologies aimed at […] prevention, characterised by a lengthy process of medical surveillance to resolve diagnostic uncertainty, which may spill over into personal identity and other areas of life’ (Timmermans and Buchbinder 2010: 419).

As the wording and definitions of the concepts suggest, previous research has favoured the illness/disease side of risk ambiguity. The concepts share an understanding of persons at risk as patients (of some kind) and, perhaps somewhat less clearly, the framing of the at‐risk status through the (chronic) illness experience. This corresponds to the thrust of the current biomedical approach to risk, which is towards the convergence of risk and disease (Aronowitz 2009). This study attempts to give more prominence to the health side of the risk ambiguity.

## Methodology

Interviewees were recruited from the (now defunct) Consumer Panel maintained for research purposes by the National Consumer Research Centre. The Panel was a register of around 1000 voluntary citizens from seven different urban areas in Finland willing to participate in the institute's research projects. Volunteers were rewarded with an annual subscription of a consumer‐themed magazine plus small gifts for each research project. The Panel was not a representative sample of the Finnish population; it consisted of relatively active consumers willing to discuss various issues that interested them.

In the recruitment letter, Panel members were invited to participate in a study of the everyday management of cholesterol risk. For practical reasons, only people from the Helsinki area were invited. The letter detailed the standard issues related to informed consent, such as background and topic of the study, use and management of the data and principles of anonymity and confidentiality. The Panel members could then volunteer to participate by responding to the invitation and filling in a background information questionnaire. This was taken to signify consent. At the start of the interview the responsibilities of the researcher and the rights of the research subject were again recapitulated. It should be noted that all the participants had received basic information on the principles of research ethics when they became Panel members.

Although the participants had various grades of cholesterol‐related issues as well as contact with medical services, they were approached not primarily as patients, but as members of the public with cholesterol issues. Both individuals with elevated cholesterol levels and those otherwise concerned about their cholesterol were targeted. I consciously also involved individuals with no objective knowledge of their cholesterol levels or a diagnosis of hypercholesterolaemia in order to capture the full spectrum of risk experience.

The background questionnaire asked about cholesterol anxiety (concerns/no concerns), cholesterol levels, use of medication, felt symptoms (if any) and standard socioeconomic information. Based on this data, individuals with an incomplete questionnaire, not concerned about their cholesterol, or with a history of heart conditions or severe symptoms (such as bypass surgery, atrial fibrillation, or pacemaker) as well as the very elderly, were excluded. The remaining individuals were then divided into four groups, and four individuals from each group were selected for interviews: (i) normal or somewhat elevated cholesterol (4.8–5.7 mmol/l), (ii) high cholesterol (5.9–6.7 mmol/l), (iii) cholesterol medication and (iv) minor cardiovascular symptoms (such as occasional arrhythmia or palpitations). Participants from Groups 3 and 4 often but not always also had somewhat elevated or high cholesterol. The aim of the grouping was to reach a good balance between homogeneity and variety among the participants.^1^ These categories are used in citations, alongside age and a pseudonym denoting gender. Differences between the groups were observed in the analysis and are reported where appropriate.

Due to the structure of the Consumer Panel, women, the middle‐aged and the elderly and the relatively well‐educated were overrepresented among those responding to the call. This bias could be rectified in the participant selection to a certain degree, but not entirely. After two selected individuals withdrew on short notice, one from each of Groups 2 and 3, I ended up conducting 14 interviews with ten women and four men, aged between 42 and 65, half with a higher and half with a lower educational degree. Although a small group, the resulting data are well suited to articulating the lay meanings attached to elevated cholesterol as a health risk.

The interview procedure combined a narrative approach with a loosely structured list of questions. The interviews began with what I called the personal ‘cholesterol story’, that is the participant's free account of their experiences with cholesterol in a loosely chronological order. Taking my cue from the story, I posed follow‐up questions about its various aspects as well as complementary questions on themes not addressed through the story. The aim of the procedure was to tease out the specific perspective from which the participant approached the cholesterol problem. In addition to the biographical information covered by the ‘cholesterol story’, major interview areas included: (i) the sources of cholesterol awareness and anxiety; (ii) the effects of cholesterol awareness or anxiety on everyday practices, habits and lifestyle aspects; (iii) ideas about the nature and effects of (elevated) cholesterol in the body; (iv) ideas about risk, health and illness and their relationship with each other. Before the interviews, the draft interview guide was tested for length, clarity and organisation with a volunteer layperson.

The interviews lasted between 71 and 107 minutes. They were tape recorded and transcribed verbatim by a professional company. All transcripts were imported into the Atlas/TI‐program for qualitative analysis. I developed an initial code list based on the reading of one interview. An experienced research assistant then coded the same interview, suggesting amendments to the code list. We compared our coding, discussed the differences and agreed on a common list of themes and codes. The research assistant then coded the rest of the interviews. During the process, some new codes emerged and these were incorporated into the initial coding list and used throughout. After this initial coding, I identified themes related to key items in the biographical disruption model. These served as higher order themes under which codes from the initial round were assigned. At this stage, illustrative quotes were selected to highlight the themes and codes.

## Contrasting the experiences of risk and chronic illness

Michael Bury (1982, 1991) has characterised chronic illness as a ‘critical situation’, which disrupts taken‐for‐granted assumptions and structures of everyday life and calls for adjustment and adaptive measures. This biographical disruption necessitates a reframing of personal biography and identity, rearranges family relations and social networks, and often entails a lasting but temporally varying relationship with medical services. Subsequent research has amended and supplemented the framework in many ways (e.g. Richardson *et al*. 2006, Williams 2000), yet the basic idea remains salient. Bury (1991: 452‐3) identified three aspects of the chronic illness experience, which roughly correspond to the temporal trajectory of the development of a condition. I will address each of these aspects in turn, ‘starting with the initial disruption of illness, then going on to the processes of explanation and legitimation, before turning to treatment and adaptation’, discussing the ways in which the risk experience overlaps with and differs from them.

### Onset: emerging cholesterol anxiety

According to Bury (1982: 169), at the onset of the illness ‘there is the disruption of taken‐for‐granted assumptions and behaviours; the breaching of common‐sense boundaries. […] This “what is going on here” stage involves attention to bodily states not usually brought into consciousness and decisions about seeking help’. This disruption generates a process of recognition, both on the part of the affected person and his or her social surroundings, including medical actors, resulting in a diagnosis and an altered identity.

All my participants were worried about their cholesterol to a greater or lesser extent, some with more ‘objective’ reasons, others with less. This was no surprise, as it was the starting point in the recruitment. The personal cholesterol stories that initiated the interviews provided an insight into the emergence of their cholesterol anxiety. A few began by discussing cholesterol as a health issue in general. For example Hanna (63, symptoms), a keen reader of health and women's magazines, recounted that although cholesterol emerged as a topic in the 1990s, she did not take it personally in any way at that time. Although these recollections thus are insufficient without other factors to generate personal cholesterol anxiety, they point to the fact that cholesterol is a culturally recognised issue that guides our health behaviour through well‐established dietary principles concerning fat consumption. Thus, some kind of background‐level of cultural cholesterol awareness exists, which feeds into anxiety.

A few participants had a family history of high cholesterol and/or cardiovascular problems, through which they had become conscious of elevated cholesterol as a health issue. For example Niina (49, symptoms) remembered being aware as a child that her mother had high cholesterol, but it is difficult to assess whether this fact only gained salience after her own diagnosis. Nevertheless, such family connections make the cholesterol issue a bit more personal than general cultural awareness.

However, since elevated cholesterol is a symptomless condition, it was cholesterol measurements that moved the issue from speculative to tangible for the participants. All of them discussed test results which had made real the fact of elevated cholesterol and thus cardiovascular risk. The first measurement was typically a routine test, for example at a health fair, when changing jobs or at an annual health check‐up.^2^ In some cases, the person's first cholesterol levels were taken in a clinical test related to a non‐cardiovascular health condition. The tests were often entered in a casual and unreflective manner, either as part of a medical routine or on a whim in the case of instant tests offered for educative purposes at events.

Despite the prominence of testing, most of the participants found it difficult to single out a specific disruptive moment in their cholesterol history. Recalling the point in time at which they first became aware of their elevated cholesterol typically required some effort, as the first tests had taken place in the distant past, but more importantly, the results seldom produced significant reactions or adjustments at first. For many participants, learning of their condition represented only the beginning of a process of slowly intensifying cholesterol awareness and anxiety. They registered the fact that their cholesterol was elevated, but this did not initially generate much reflection or action.

The few exceptions to this pattern among my participants raises the question of stigma. For those the test results came as a surprise, which could elicit strong, emotional reactions. Pauliina (62, somewhat elevated cholesterol) recalled the initial ‘shock’ she felt upon learning of her elevated cholesterol when taking a test with two colleagues: ‘these two women got a clean bill of health, I think [they measured] five point something, while I had seven point six or something like that. I felt like a bad person, a failure and was deeply ashamed’. The reaction of another participant, Ingrid (50, somewhat elevated cholesterol) to her test results hints at the nature of the shock. She was surprised by her level, since she ‘had been rather sporty and lived quite healthily all‐round’. Thus, the feelings of shock and surprise were generated if the fact of elevated cholesterol was in conflict with their self‐image at the time: being a young person of normal weight, following a healthy lifestyle. It seems that the shock primarily concerned not the (risk of) illness itself, but the discrediting of personal habits and its moral implications. Even for these participants, cholesterol soon returned backstage in their lives, after some passing attempts at lifestyle modification.

Looking forward at the participants’ illness trajectories does not change the overall picture. The cholesterol issue typically became somewhat more pressing with age, reflecting the fact that both cholesterol levels and frequency of testing tend to increase when people get older. At this stage, minor cardiovascular symptoms and/or medication may enter the scene. Yet medical contact or starting a drug regime did not emerge as specific turning points in the participants’ biographies. The symptoms were typically vague and had not produced a definitive diagnosis. Some participants who had indicated having minor symptoms in the background questionnaire bypassed or downplayed them during the interview. Medication was considered slightly more of an alteration. Generally, the participants were critical of drugs. Fear of side‐effects was typically cited as justification, but the moral valence of drugs also seemed to play a major role. According to the current treatment guidelines of hypercholesterolaemia, a drug regime is only initiated when efforts to normalise cholesterol levels through lifestyle modification have been unsuccessful. Thus, medication could be interpreted as a sign of failure in rectifying elevated cholesterol through a change of habits, that is a discrediting of willpower and moral integrity on the personal level. Such reasoning is evident in Arto's (44, somewhat elevated cholesterol) story about his cousin, ‘who measures 170 cm and weights 120 kg and is on diabetes, cholesterol and hypertension medications’. Arto considers this ‘very negative’, since the cousin ‘could get rid of the medications if he lost 40 kilos and exercised a bit more’. However, ’if [elevated cholesterol] can't be rectified by lifestyle and food habit [changes], then it's ok [to take drugs]’, according to him.

### Explanation and legitimation: assessing personal habits and lifestyle

The second stage in Bury's (1982: 169) model, when the illness has become visible, involves ‘a fundamental re‐thinking of the person's biography and self‐concept’. The seemingly arbitrary disruptive event needs to be explained and given meaning. When answering Why me? Why now? questions ‘individuals not only wish to gain a measure of control over their condition by finding explanations that make sense in terms of their life circumstances and biographies, but they also wish to establish a proper sense of perspective about the condition, and re‐establish credibility in the face of the assault on self‐hood which is involved’ (Bury 1991: 455‐6). Thus, the individuals want to legitimate their new status for themselves and others, ‘to maintain a sense of personal integrity, and reduce the threat to social status, in the face of radically altered circumstances’ (Bury 1991: 456).

As discussed above, in risk conditions the circumstances are not radically altered; the change is slow and phased. The elevated cholesterol itself did not usually represent an assault on the self and initiate a fundamental rethinking of the person's biography and self‐concept, except for among a few participants whose cholesterol anxiety started at a relatively young age and involved an element of surprise. For most, elevated cholesterol was a slowly emerging and somewhat vague concern that was often qualified by proportioning it to other, more pressing health problems or risk factors. In these cases, familial relations and biography could play a role. For example Anna's (61, symptoms) family history made her more worried about cancer than CVD, despite having a total cholesterol of 6.3 mmol/l. Several participants mentioned diabetes as a more frightening disease than CVD, with unpleasant symptoms and consequences such as eye or feet damage. Similarly, the specific risk factors associated with diabetes, especially high blood sugar, were considered more severe than elevated cholesterol. Perhaps the image of CVD as a largely unforeseeable disease leading to sudden death (cardiac arrest), rather than as a chronic condition with debilitating effects, played a role in this stance.

The participants utilised a very narrow framework when explaining their elevated cholesterol. Their explanations were based on the perceived close connection between personal habits and cholesterol levels, while, unlike in other studies concerning CHD risk, aspects of the broader social and personal context (e.g. Davison *et al*. 1991, Lupton and Chapman 1995) or references to ‘chance’ or ‘luck’ (Davison *et al*. 1992) were less prominent. Although the participants were aware on an abstract level of the role that chance, for example plays in probabilistic risk, they did not utilise such possibilities when explaining their personal situation. Participants believed strongly that their cholesterol levels were ultimately in their own hands, and that changes in habits were directly reflected in the levels. For some, this belief had resulted from successful lifestyle modification, whereas others were convinced that they could lower their levels if they became concerned enough to put in the proper effort: ‘I have done as much as I can manage. And I know I could do more, if I [lived] very puritanically, but at the moment I don't consider the risk large enough’ (Outi, 47, somewhat elevated cholesterol). Some who struggled with their cholesterol, castigated themselves for not being strict enough in their diet or other health‐related habits: ‘I know what I should do. But then in practice things get left undone’ (Mia, 55, medication).

It is also remarkable how the participants seldom resorted to blame‐shifting to alleviate moral responsibility for their abnormal cholesterol. They only rarely amended the dominant explanatory framework with other factors outside their control, such as heredity, metabolic idiosyncrasies or life‐situation‐related stress. Such factors were evoked when the expected correlation between lifestyle characteristics and cholesterol levels did not hold. A healthy lifestyle leading to normal cholesterol and an unhealthy lifestyle leading to elevated cholesterol were expected situations and did not warrant comment. My material had no cases of a decidedly unhealthy lifestyle but normal cholesterol; the participants were people who were worried about their cholesterol and conscientious about their healthcare practices. Only when the levels remained high despite the participants’ best efforts to modify them through lifestyle changes were other explanations sought. Elina (60, high cholesterol) had experimented with cutting out cheese from her diet and using Benecol, a cholesterol‐lowering bread spread, as well as following her eating with a food diary, but to no avail: ‘whatever I do or don't do, the level keeps rising’. Disillusioned, she had begun to speculate on hereditary factors causing her predicament, since her mother had died of coronary thrombosis at the age of 38. This pattern again highlights how deeply ingrained personal responsibility for health and lifestyle as the privileged arena of change is in Western culture. Sachs (1995) and Saukko *et al*. (2012) noted similar shifts from causal theories based on lifestyle choices to other explanations in relation to cholesterol, as did Lawton *et al*. (2008) in relation to diabetes.

Legitimation follows a similar pattern. The sole fact of elevated cholesterol did not generate a need for legitimation, unless the news came as a surprise or shock. Elevated cholesterol is a hidden condition, without felt symptoms or outward signs of illness, and thus does not compromise a person's social status. The need for legitimation increased if the participant had to begin a drug regime or if symptoms emerged. Cholesterol levels were regarded as an indicator of the healthiness of personal habits, especially eating and exercising and problems in controlling the levels hinted at moral failure. In all cases, the crucial issue was how to fight the discrediting of personal habits affected by elevated cholesterol and to sustain an image of a responsible subject of health care, in both their own eyes and in those of others (cf. Riessman 1990).

### Adaptive responses: adjusting habits, seeking medical contact

The third stage in the chronic illness experience model covers the adaptive responses to disruption. Bury (1991) distinguishes between coping, strategy and style when discussing these responses. ‘Coping’ refers to ‘the cognitive processes whereby the individual learns how to tolerate or put up with the effects of illness’ (Bury 1991: 460). In the case of cholesterol risk, the effects are diffuse, as no symptoms exist. Therefore, individuals with elevated cholesterol do not really need to ‘maintain a sense of value and meaning in life’ (Bury 1991: 461). However, what they do need to do is cope with the often distant possibility of cardiovascular illness. In previous discussion I have characterised the mode of being towards this possibility as cholesterol anxiety, a slowly emerging awareness and somewhat vague concern, which is either supressed as untimely or irrelevant, qualified by more pressing health issues, or embraced as a motivator for lifestyle changes.

‘Strategy’ points to ‘the actions people take, or what people do in the face of illness, rather than the attitudes people develop’ (Bury 1991: 461). This strategic management of illness, ‘from the person's viewpoint, means not only the skilful manipulation of social settings and appearances to minimise the impact of illness on interaction but also the attempt to mobilise resources to advantage, and the setting of realistic goals in order to maintain everyday life’ (Bury 1991: 462).

Two key management strategies emerged from the interviews. One was lifestyle monitoring and modification. Corresponding to the firm belief in the close links between personal habits and cholesterol levels discussed above, individuals responded to their elevated levels by reviewing their eating habits and increasing the amount they exercised. These attempts were more or less intermittent and conditioned by the severity of the problem. In many cases they had been triggered by cholesterol measurements, which often started a period of concern and activity (cf. Gillespie 2012). Although conscious and purposeful attempts at lifestyle modification were often short lived, the participants felt that they could result in permanent changes by introducing new healthy habits or erasing old unhealthy ones. These courses could easily be followed within the limits of everyday life, and only seldom required manipulation of social settings (e.g. when following a certain diet clashed with social demands). Beyond such bursts of lifestyle modification exists a more continuous monitoring of personal habits according to established cultural beliefs and values concerning healthy eating, exercising, etc.

The other management strategy was rationing medical surveillance. Since elevated cholesterol is an asymptomatic condition, measurements are crucial in assessing personal status at different stages of the ‘risk trajectory’. The ways in which the participants approached measuring varied greatly. Some actively sought regular monitoring, as they considered ‘numerical facts’ motivating when embarking on lifestyle modification (Matti, 49, high cholesterol) and helpful when assessing their success. Others avoided testing as a way of coping: ‘Yes it has been monitored, but [now] I think I won't say “take it” anymore, as it just keeps rising all the time’ (Elina, 60, high cholesterol). Most of the participants were, however, satisfied with the occasional cholesterol measuring offered by medical services. Those with symptoms or on medication were of course tested more regularly. Thus, medical contact is not necessarily primarily situated at the onset of the illness trajectory: here it was a key strategy in adaptive responses to the risks affected by elevated cholesterol.

Lastly, ‘style’ refers to the class‐specific variations in the ways in which ‘people respond to, and present important features of their illness or treatment regimens’ (Bury 1991: 462). Although my interviewees represented various levels of education, they were remarkably uniform in their adaptive responses. This likely reflects their nature, and such a small study is not ideal in determining differences in adaptive styles.

## Patients or ‘health citizens’?

The analysis shows that the everyday management of cholesterol anxiety of my participants did not conform to Bury's (1982, 1991) biographical disruption framework, despite its popularity for modelling the ‘liminal’ experience of risk.

Using the framework typically carries with it the assumption that persons at risk are patients (of some kind). Yet my participants were rather reluctant to accept patient status. This became evident when I asked them how they related the risky condition of elevated cholesterol to health and illness, both abstractly and in terms of their own condition. On the abstract level, the participants identified several criteria for when elevated cholesterol signified illness: if it caused ‘proper’ illness with symptoms; if it was accompanied by other, visible signs considered to indicate health problems, such as overweight; if it was medicated; and if it affected capacity to function, e.g. work and motion. Several participants stressed the importance of subjective feelings of well‐being, which were considered decisive in judging the health status of an individual.

However, in relation to their own condition, the participants used the criteria to *create distance* from patient status. For those with only somewhat elevated cholesterol this was easy, since they could refer to all criteria simultaneously: no visible signs, symptoms, medication or diminished capacities equals health. For those with high cholesterol the issue was more tangible, yet the stance was the same. This is how Matti (49, high cholesterol) reacted to a question concerning the status of a person with 6.5 mmol/l cholesterol (his own level): ‘He is on his way to becoming an ill person, but I don't personally feel ill now. There's no such measurable quantity that says I'm ill, save for the level of 6.5. It's not like taking your temperature, 37 or more and you have a fever, less and you are evidently healthy’. One participant in this group introduced the idea of a personal cholesterol level, which is higher than average but still normal for her, thus implicitly situating her among the healthy: ‘Nowadays I think that my heredity is such that it [cholesterol] is just a bit higher than that of some others’ (Pauliina, 63, high cholesterol).

For those with medication or minor cardiovascular symptoms, distancing was more difficult in principle, because they fulfilled one of the criteria. However, they also did not readily accept the notion of illness. ‘I feel quite healthy’, said Anna (63, symptoms), who was suffering from arrhythmia and had 6.3 mmol/l total and 4.1 mmol/l LDL cholesterol. Such statements were typical among all the participants, also those in Groups 3 and 4. The various criteria identified were used as a cascading set of justifications for rejecting illness. While those who had experienced minor heart‐related symptoms might appeal to missing medication or an undiminishing capacity to act, those who were on medication could refer to the absence of symptoms or enduring vigorousness as grounds for being healthy, as in the following excerpt:

Mia (55, medication) I think a person can have even high cholesterol, but still be healthy.

AUTHOR Okay. In which case is she then no longer healthy?

Mia If she has been diagnosed with an illness.

AUTHOR Okay.

Mia But, for example also a very overweight person can be healthy, if she exercises and has no confirmed diseases.

The only exception to this pattern was the participant with diabetes, although not due to his CVD‐related risk factors, but because of his confirmed diagnosis of diabetes.

Thus, the participants, while recognising the peculiar position of at‐risk states between health and disease, were clear in their judgement that for them personally, the risk did not (yet) represent illness. Instead of patients‐in‐waiting suffering from proto‐illness, the participants presented themselves as ‘chronically healthy individuals’ (Varul 2010: 86), who are actively trying to *avoid* becoming patients. This aspiration was fulfilled by engaging in various everyday healthcare practices. The bulk of the interviews addressed issues concerning food practices and exercising habits, not medical surveillance and treatments. The key concern for the participants was not how to treat cholesterol risk, but how to maintain their health.

## Discussion and conclusion

The pattern presented differs markedly from other studies on the risk experience of elevated cholesterol. For example in her study on middle‐aged men enrolled in a cholesterol screening and health education regime, Sachs (1995; for more examples see Crinson *et al*. 2007: 366‐7) found that the tests had clearly disruptive effects on some of her participants, although not on all of them. Knowledge of elevated cholesterol could generate ‘profound anxiety’ and ‘mortal fear’, which were countered with attempts to change one's lifestyle. For example in the case of one of Sachs’ participants ‘his whole life was changed by the check‐up. […] The biomedical way of ordering his world of experience filtered into his whole existence. It infected his wife and children and his overall network of friends’, and this led to increasing isolation. (Sachs 1995: 512)

Similarly, Gillespie (2015) identified significantly altered circumstances and wide‐reaching adaptive responses among individuals obtaining an at‐risk status through routine cholesterol or PSA screening. According to him, the main social effects of this situation are increased medical contact (including medicine use), a restructuring of everyday routines (including lifestyle effects, e.g. diet and physical activity) and altered social relationships (including family, parents and colleagues). Referring to Rosenberg's concept, he suggests ‘that there is a corresponding illness experience that results from a diagnosis of a proto‐disease. The participants were not managing the symptoms of disease, but were managing the consequences of a perceived illness in the absence of symptoms’. (Gillespie 2015: 984)

What could account for this difference? One factor might be the fact that this is a small study with a selected group of participants who are likely to be more health conscious than Finns on average. This might have led them to accentuate health management and coping instead of patient behaviour. Another factor is the nature of elevated cholesterol as a condition that is both connected to lifestyle choices and treatable by effective medications.^3^ Other risk factors, with less clear‐cut behavioural pathways, might generate more patient talk, while conditions with no such effective treatments might elicit reactions that are more biographically disruptive. This explanation, however, is weakened by the empirical findings presented above that highlight elevated cholesterol as a critical situation with disruptive effects.

Still another explanation might be the method of recruiting the research subjects and the (consequent) framing of the issue. Previous studies have often recruited research subjects from medical contexts, and the literature review presented suggested that persons at risk have typically been defined as patients (of some kind) and the risk experience modelled after the chronic illness experience. This study differed from this paradigm, which might have influenced the aspects of the risk experience participants highlighted in their responses: instead of negotiating their patient status and relationship with medical services, they assessed their healthcare practices as responsible ‘health citizens’. This explanation posits the co‐existence of two types of discourse, clinical and public health (Saukko *et al*. 2012), which are activated depending on the context and stimulus. The benefit of this explanation is that it would solve the contradiction between different research results: in the clinical discourse the at‐risk status is equated with illness (Kreiner and Hunt 2014; Gillespie 2015) or perceived as imperfect and ambivalent compared to the medical norm of a definite diagnosed disease with clear therapeutic pathways (Scott *et al*. 2005), whereas the public health discourse accentuates self‐care practices in the everyday context, personal responsibility and overall health and well‐being. These various explanations should be the subject of further research.

In summary, this study cautions against the privileging of the illness/disease side of the risk ambiguity. Risk consciousness is not restricted to the medical realm; it has been culturally generalised, structuring contemporary everyday practices. Being at risk generates, in addition to proto‐illness‐behaviour, an intensification of healthcare practices, many of which consist of managing the lifestyle aspects of health that have been linked to various risk factors for common chronic diseases. The pervasiveness of risk, its tendency to cover each and every one of us, and its relationship with mundane decisions concerning eating, drinking, exercising, etc. mean that it is essential that we also study risk experiences before and beyond the medical realm.

## Notes

1 One participant did not report that he was suffering from diabetes. Moreover, some had experienced cardiovascular symptoms but did not report these in the questionnaire, whereas others with reported symptoms did not discuss them during the interview.

2 In Finland, many employers offer new employees a health check‐up, whereas some municipalities have check‐ups for citizens in specific age groups (e.g. 40‐year‐old males in Helsinki).

3 This point was raised by one of the anonymous reviewers. A further explanation might be cultural differences in screening practices. Perhaps in other countries, elevated cholesterol is more strongly medicalised than in Finland, with even minor abnormalities leading to intense monitoring and consequently generating patient identities?

## References

[shil12866-bib-0001] Angus, J. , Evans, S. , Lapum, J. , Rukholm, E. , *et al* (2005) ‘Sneaky disease’: the body and health knowledge for people at risk of coronary heart disease in Ontario, Canada, Social Science and Medicine, 60, 9, 2117–28. 15743659 10.1016/j.socscimed.2004.08.069

[shil12866-bib-0002] Armstrong, D. (1995) The rise of surveillance medicine, Sociology of Health & Illness, 17, 3, 393–404.

[shil12866-bib-0003] Aronowitz, R.A. (2009) The converging experience of risk and disease, The Milbank Quarterly, 87, 2, 417–42. 19523124 10.1111/j.1468-0009.2009.00563.x PMC2728027

[shil12866-bib-0004] Berlivet, L . (2005) ‘Association or Causation?’ The Debate on the Scientific Status of Risk Factor Epidemiology, 1947—c. 1965 In Berridge V (ed) Making Health Policy: Networks of Research and Policy After 1945. Amsterdam, New York: Rodopi. 16212726

[shil12866-bib-0005] Bunton, R. and Burrows, R. (1995) Consumption and health in the ‘epidemiological’ clinic of late modern medicine In Bunton R., Nettleton S. and Burrows R. (eds) The Sociology of Health Promotion: Critical Analyses of Consumption, Lifestyle and Risk. London and New York: Routledge.

[shil12866-bib-0006] Bury, M. (1982) Chronic illness as biographical disruption, Sociology of Health & Illness, 4, 2, 167–82. 10260456 10.1111/1467-9566.ep11339939

[shil12866-bib-0007] Bury, M. (1991) The sociology of chronic illness: a review of research and prospects, Sociology of Health and Illness, 13, 4, 451–68.

[shil12866-bib-0008] Crawford, R. (1980) Healthism and the medicalization of everyday life, International Journal of Health Science, 19, 365–88. 10.2190/3H2H-3XJN-3KAY-G9NY 7419309

[shil12866-bib-0009] Crinson, I. , Shaw, A. , Durrant, R. , De Lusignan, S. , *et al* (2007) Coronary heart disease and the management of risk: patient perspectives of outcomes associated with the clinical implementation of the National Service Framework targets, Health, Risk & Society, 9, 4, 359–73.

[shil12866-bib-0010] Davison, C. , Davey, Smith G. and Frankel, S. (1991) Lay epidemiology and the prevention paradox: the implications of coronary candidacy for health education, Sociology of Health & Illness, 13, 1, 1–19.

[shil12866-bib-0011] Davison, C. , Frankel, S. and Davey‐Smith, G. (1992) The limits of lifestyle: re‐assessing ‘fatalism’ in the popular culture of illness prevention, Social Science and Medicine, 34, 6, 675–85. 1574735 10.1016/0277-9536(92)90195-v

[shil12866-bib-0012] Davison, C. , Macintyre, S. and Davey‐Smith, G. (1994) The potential social impact of predictive genetic screening for susceptibility to common chronic diseases, A Review and Proposed Research Agenda, Sociology of Health & Illness, 16, 3, 340–71. 11660089 10.1111/1467-9566.ep11348762

[shil12866-bib-0013] Dumit, J. (2012) Drugs for Life: How Pharmaceutical Companies Define our Life. Durham and London: Duke University Press.

[shil12866-bib-0014] Finkler, K. (2001) The Kin in the Gene, The Medicalization of Family and Kinship in American Society, Current Anthropology, 42, 2, 235–63. 14992215

[shil12866-bib-0015] Garrety, K. (1997) Social worlds, actor‐networks and controversy: the case of cholesterol, dietary fat and heart disease, Social Studies of Science, 27, 5, 727–73. 11619512 10.1177/030631297027005002

[shil12866-bib-0016] Gillespie, C. (2012) The experience of risk as ‘measured vulnerability’: health screening and lay uses of numerical risk, Sociology of Health & Illness, 34, 2, 194–207. 21848989 10.1111/j.1467-9566.2011.01381.x

[shil12866-bib-0017] Gillespie, C. (2015) The risk experience: the social effects of health screening and the emergence of a proto‐illness, Sociology of Health & Illness, 37, 7, 973–87. 25912148 10.1111/1467-9566.12257

[shil12866-bib-0018] Giroux, E. (2013) The framingham study and the constitution of a restrictive concept of risk factor, Social History of Medicine, 26, 1, 94–112.

[shil12866-bib-0019] Greaves, D. (2000) The creation of partial patients, Cambridge Quarterly of Health Care Ethics, 9, 1, 23–33. 10721467 10.1017/s0963180100001043

[shil12866-bib-0020] Greene, J.A. (2007) Prescribing by Numbers: Drugs and the Definition of Disease. Baltimore: Johns Hopkins University Press.

[shil12866-bib-0021] Hansen, E. and Easthope, G. (2007) Lifestyle in Medicine. London and New York: Routledge.

[shil12866-bib-0022] Hoel Felde, L.K . (2010) I take a small amount of the real product’: elevated cholesterol and everyday medical reasoning in liminal space, Health: An Interdisciplinary Journal, 15, 6, 604–19. 10.1177/1363459310364160 21177710

[shil12866-bib-0023] Hughes, M. (1994) The risks of lifestyle and the diseases of the civilization, Annual Review of Health Social Sciences, 4, 1, 57–78.

[shil12866-bib-0024] Jensen, T.Ø. (1994) The political history of Norwegian nutrition policy In Burnett J. and Oddy D.J. (eds) The Origin and Development of Food Policies in Europe. London and New York: Leicester University Press.

[shil12866-bib-0025] Kenen, R.H. (1994) The human genome project: creator of the potentially sick, potentially vulnerable and potentially stigmatized? In Robinson I. (ed) The Consequences of Life and Death Under High Technology Medicine. Manchester: University of Manchester Press.

[shil12866-bib-0026] Kenen, R.H. (1996) The at‐risk health status and technology: a diagnostic invitation and the ‘gift’ of knowing, Social Science and Medicine, 42, 11, 1545–53. 8771637 10.1016/0277-9536(95)00248-0

[shil12866-bib-0500] Kenen, R. , Ardern‐Jones, A. and Eeles, R. (2003) Living with chronic risk: healthy women with a family history of breast/ovarian cancer, Health, Risk & Society, 5, 3, 315‐31.

[shil12866-bib-0027] Kleinman, A . (1988) The Illness Narratives. Suffering, Healing, and the Human Condition. New York: Basic Books.

[shil12866-bib-0028] Kokko, S. and Räsänen, L. (1997) Nordic nutrition policies, Finland Case Study, Nutrition Reviews, 55, 11, S21–S28. 9420458 10.1111/j.1753-4887.1997.tb01570.x

[shil12866-bib-0029] Konrad, M. (2003) Predictive genetic testing and the making of the pre‐symptomatic person: prognostic moralities amongst Huntington's‐affected families, Anthropology & Medicine, 10, 1, 23–49. 26867065 10.1080/13648470301269

[shil12866-bib-0030] Kreiner, M.J. and Hunt, L.M. (2014) The pursuit of preventive care for chronic illness: turning healthy people into chronic patients, Sociology of Health & Illness, 36, 6, 870–84. 24372285 10.1111/1467-9566.12115 PMC4074448

[shil12866-bib-0031] La Berge, A.F. (1998) How the ideology of low fat conquered America, Journal of the History of Medicine and Allied Sciences, 63, 2, 139–77. 10.1093/jhmas/jrn001 18296750

[shil12866-bib-0032] Lawton, J. , Peel, E. , Parry, O. and Douglas, M. (2008) Shifting accountability: a longitudinal qualitative study of diabetes causation accounts, Social Science and Medicine, 67, 1, 47–56. 18440113 10.1016/j.socscimed.2008.03.028

[shil12866-bib-0033] Lupton, D. (1993) Risk as moral danger: the social and political functions of risk discourse in public health, International Journal of Health Services, 23, 3, 425–35. 8375947 10.2190/16AY-E2GC-DFLD-51X2

[shil12866-bib-0034] Lupton, D. and Chapman, S. (1995) A healthy life‐style might be the death of you ‐ discourses on diet, cholesterol control and heart‐disease in the press and among the lay public, Sociology of Health & Illness, 17, 4, 477–94.

[shil12866-bib-0035] Madarász, J. (2010) Prävention chronischer Herz‐Kreislauf‐Krankheiten BRD, DDR und Großbritannien im Vergleich, 1945–1990, Prävention und Gesundheitsförderung, 5, 4, 313–8.

[shil12866-bib-0036] Nestle, M. (2007) Food Politics. How the Food Industry Influences Nutrition and Health. Berkeley: University of California Press.

[shil12866-bib-0037] Oppenheimer, G.M. (2005) Becoming the framingham study 1947—50, American Journal of Public Health, 95, 4, 602–10. 15798116 10.2105/AJPH.2003.026419 PMC1449227

[shil12866-bib-0038] Paquette, M.‐C. (2005) Perceptions of healthy eating: state of knowledge and research gaps, Canadian Journal of Public Health, 96, S16–9.16042159

[shil12866-bib-0039] Parascandola, M. (2004) Skepticism, statistical methods, and the cigarette: a historical analysis of a methodological debate, Perspectives in Biology and Medicine, 47, 42, 244–61. 15259206 10.1353/pbm.2004.0032

[shil12866-bib-0041] Povey, R. , Conner, M. , Sparks, P. , James, R. , *et al* (1998) Interpretations of healthy and unhealthy eating, and implications for dietary change, Health Education Research, 13, 2, 171–83. 10181016 10.1093/her/13.2.171

[shil12866-bib-0042] Richardson, J.C. , Ong, B.N. and Sim, J. (2006) Is chronic widespread pain biographically disruptive?, Social Science and Medicine, 63, 6, 1573–85. 16697511 10.1016/j.socscimed.2006.03.040

[shil12866-bib-0043] Riessman, C.K. (1990) Strategic uses of narrative in the presentation of self and illness—a research note, Social Science and Medicine, 30, 11, 1195–200. 2360055 10.1016/0277-9536(90)90259-u

[shil12866-bib-0044] Rose, G. (1981) Strategy of prevention ‐ lessons from cardiovascular disease, British Medical Journal, 2, S2, 1847–51. 10.1136/bmj.282.6279.1847 PMC1506445 6786649

[shil12866-bib-0045] Rosenberg, C.E. (1997) Banishing Risk: Continuity and Change in the Moral Management of Disease In Brandt A.M. and Rozin P. (eds) Morality and Health. New York and London: Routledge.

[shil12866-bib-0046] Rothstein, W.G. (2003) Public Health and the Risk Factor. A History of an Uneven Medical Revolution. Rochester and Woodbridge: University of Rochester Press.

[shil12866-bib-0047] Sachs, L. (1995) Is there a pathology of prevention?, The Implications of Visualizing the Invisible in Screening Programs, Culture, Medicine and Psychiatry, 19, 4, 503–25. 10.1007/BF01379400 8838297

[shil12866-bib-0048] Sachs, L. (1996) Causality, responsibility and blame – Core issues in the cultural construction and subtext of prevention, Sociology of Health & Illness, 18, 5, 632–52.

[shil12866-bib-0049] Saukko, P.M. , Farrimond, H. , Evans, P.H. and Qureshi, N. (2012) Beyond beliefs: risk assessment technologies shaping patients’ experiences of heart disease prevention, Sociology of Health & Illness, 34, 4, 560–75. 22017639 10.1111/j.1467-9566.2011.01406.x

[shil12866-bib-0050] Scott, S. , Prior, L. , Wood, F. and Gray, J. (2005) Repositioning the patient: the implications of being ‘at‐risk’, Social Science and Medicine, 60, 8, 1869–79. 15686817 10.1016/j.socscimed.2004.08.020

[shil12866-bib-0051] Sulik, G.A. (2009) Managing biomedical uncertainty: the technoscientific illness identity, Sociology of Health & Illness, 31, 7, 1059–76. 19619153 10.1111/j.1467-9566.2009.01183.x

[shil12866-bib-0052] Talley, C. , Kushner, H.I. and Sterk, C.E. (2004) Lung cancer, chronic disease epidemiology, and medicine, 1948–1964, Journal of the History of Medicine and Allied Sciences, 59, 3, 329–74. 15270334 10.1093/jhmas/jrh088

[shil12866-bib-0053] Timmermans, S. and Buchbinder, M. (2010) Patients‐in‐waiting: living between sickness and health in the genomics era, Journal of Health and Social Behavior, 51, 4, 408–23. 21131618 10.1177/0022146510386794

[shil12866-bib-0054] Varul, M.Z. (2010) Talcott parsons, the sick role and chronic illness, Body & Society, 16, 2, 72–94.

[shil12866-bib-0055] WHO (2018) Health topics: Risk factors. Available at http://www.who.int/topics/risk_factors/en/ (Last accessed 25 June 2018).

[shil12866-bib-0056] Williams, S.J. (2000) Chronic illness as biographical disruption or biographical disruption as chronic illness?, Reflections on a Core Concept, Sociology of Health and Illness, 22, 1, 40–67.

